# Case of Acute Inflammation of the Psoac Muscles, with Observations

**Published:** 1817-08

**Authors:** James Kennedy

**Affiliations:** Dunning, Perthshire


### *Case of Acute Inflammation of the Psoac Muscles, with Observations.*

By James Kennedy, M. D. Dunning, Perthshire.

THE occurrence of acute inflammation of the psoac muscles has never, as far as my acquaintance with medical literature extends, been regularly described by nosologists; nor do I remember to have seen its characters correctly defined in the writings of practical men. Without desiring to attach any high practical importance to the distinction, I am induced to believe that some of the phænomena, which mark the existence of genuine acute inflammation of the psoac muscles may, not obscurely, be traced in a sketch of the pathognomonic signs which the following case exhibited.

On the morning of the 10th of December, 1816, I first visited T. F. a stout active man, aged twenty-five, and of a strongly-marked sanguineous temperament. He stated that, about eight days previously to this period, he had been seized, after violent exercise followed by exposure to cold, with most severe and constant pain in the lumbar region. It was much aggravated by motion, particularly of the lower extremities: for two days, it grew progressively worse. Confinement to bed, however, and a full dose of Epsom salts, so far relieved the young man's complaint on the eighth, as to allow him to walk out and shoot on the river Earne. But this premature exertion, aided by the influence of cold air, brought on a return of the disease with increased violence. He was unable to regain home and had lain upwards of an hour on the frozen ground ere he was discovered by his friends.

Duly apprized of these circumstances, I proceeded to investigate the precise seat and character of my patient's malady. The presence of synocha was announced by the state of the abdominal, thoracic, and cerebral organs; of the tongue, mouth, and cutaneous surface. The circulating system, however, did not display a proportionate disturbance. The pulse was full, regular, strong, and sluggish; sixty in the minute. The strokes of the radial and inguinal arteries were precisely synchronous. Twice, during the last twenty-four hours, a shivering fit had been experienced; but it was, in neither case, violent or long continued. The pain of the lumbar region was deep seated, incessant, lancinating, and throbbing. My patient described it as occupying a situation within his body, and causing a sensation as though some very hot substance were applied to the internal surface of his loins. Pressure on the contiguous parts produced neither pain nor irritation. The trunk could not be inclined forwards without sensations of distress. Rotation of the thighs, particularly of the right, was intolerable. A position on the left side, with the shoulders moderately inclined forward, and the knees bent, was found the most easy; and any attempt to change it induced general feelings of anguish and disquietude.

*Treatment.* The indications in this case were obvious. To subdue the inflammatory action of the muscular structure of the lumbar region was the first and most important. Upon this principle, the following plan was instituted.

Forty ounces of *blood* were drawn from the arm by a large orifice, without inducing syncope. The operation was followed by a distinct mitigation of the pain.

A drastic nauseating *purgative*, composed of twelve grains of submuriate of mercury, ten of ipecacuanha, and compound scammony powder, and a little aromatic powder, was immediately administered, and followed, in six hours, by an ounce of sulphate of magnesia.

To the whole lumbar region, previously washed with hot vinegar, the application of a *blister* was directed as soon as the cathartic should have operated. At the same time, a liberal use of *diluent drinks, abstemious regimen,* and *pediluvia,* were recommended.

On the following day, I found that the powder had acted briskly both on the stomach and bowels. The blister had risen well in eight hours, without affecting the urinary organs, and was then removed; the vesicated surface being dressed in the usual manner. The local pain was now still farther diminished, and the expression of my patient's countenance improved. The tongue and mouth presented a naturally moist appearance. The blood, drawn yesterday, shewed an excessive proportion of serum, and a coagulum covered with a strong buffy coat. The pulse was now eighty; full, strong, and regular. The disorder of respiration had considerably subsided. The cutaneous surface was restored to a healthy condition. The alvine discharges were of a greenish colour, and highly offensive.

Thirty ounces of blood were again taken from the arm. After this, the patient could move his limbs and trunk without experiencing oppression or restraint. I, moreover, directed a diaphoretic mixture, composed of solution of acetate of ammonia and an aromatic, to be taken every three hours; and a dose of castor oil at bed-time.

All the urgent symptoms were, on the third day, greatly relieved. A moderate diaphoresis had prevailed during the preceding night. Abstraction of blood, however, seemed to be still indicated. Twenty-four ounces were, therefore, again drawn, in a full stream, from the arm. The man was now permitted to discontinue his former medicines, with the exception of occasional small doses of sulphate of magnesia.

Convalescence proceeded favourably. On the 16th of the month, my patient was able to quit his bed for several hours. His appetite was keen; his digestion active; his strength greatly renovated. His pulse, sixty-four in the minute, was tranquil and equable. This probably constituted the ratio of its natural velocity. In four days more he resumed his ordinary occupations.

*Observations.* Having pronounced this to be a case of acute inflammation of the psoac muscles, I may, perhaps, be allowed cursorily to retrace and illustrate the pathognomonic signs which led to the adoption of, and seem to authorize, this opinion. The phænomena, which serve to distinguish this affection from other diseases to which it betrays an affinity, are, if I mistake not, tolerably well marked, and susceptible of clear discrimination.

With *active inflammation of the peritoneal membrane, or of any of the hollow or glandular organs occupying the abdominal cavity,* my patient's malady could not, on contemplating the seat of the pain, and the general character of his symptoms, be readily confounded.

There were, in his case, no vomiting; no pain or numbness of either inferior extremity in the course of the anterior crural nerve; no micturition, dysury, nor testicular retraction — symptoms which unequivocally characterize *inflammation, or irritative action of the kidney.*

Lastly. The present variety of inflammatory affection widely differs from *lumbar rheumatism,* as exhibiting none of the tension, smoothness, redness, and increased sensibility of the affected surface; none of the abundant perspiration; none of the pain shooting down into the sacral bone, with nocturnal exacerbations, and of that moderate degree of febrile excitement, which signalize the existence of the latter disease.

How the inert condition of the circulating system, observed in this man's case, is to be explained, I profess myself unable to determine. The character of his disease was evidently that of phlegmonous inflammation. Upon considering the situation occupied by the pain, and the aggravating influence exercised upon it by the action of a certain set of muscles, and particularly of those which rotate the femur, the attentive reader will scarcely hesitate to regard, with me, the *psoas magnus muscle* as having constituted, in this instance, the principal, if not the exclusive, seat of the inflammatory action.

Postscript.—*March* 29, 1817. This morning, I have examined the subject of the preceding case, in order to ascertain the natural condition of the circulating system. He is now free from the influence of any apparent cause, whereby the action of the heart may be excited or depressed. In fact, he exhibits the appearances of perfect health. His pulse is beating *fifty-seven strokes in the minute,* strong, full, and soft. Shall we then consider the absence of derangement of the arterial system as a diagnostic sign of acute inflammation of the psoac muscles? This, I am well aware, is a proposition which must be subjected, ere it is admitted, to the test of rigorous and reiterated observation. There are some individuals, whose pulse, from certain peculiarities of constitution, never rises above the standard of health, even in the most violent attacks of active inflammatory disease.

